# Enhancing KCC2 function counteracts morphine-induced hyperalgesia

**DOI:** 10.1038/s41598-017-04209-3

**Published:** 2017-06-20

**Authors:** Francesco Ferrini, Louis-Etienne Lorenzo, Antoine G. Godin, Miorie Le Quang, Yves De Koninck

**Affiliations:** 1 Department of Veterinary Sciences, University of Turin, Turin, Italy; 2 CERVO Brain Research Centre, Institut universitaire en santé mentale de Québec, Québec, Canada; 3 Department of Psychiatry and Neuroscience, Université Laval, Québec, Canada

## Abstract

Morphine-induced hyperalgesia (MIH) is a severe adverse effect accompanying repeated morphine treatment, causing a paradoxical decrease in nociceptive threshold. Previous reports associated MIH with a decreased expression of the Cl^−^ extruder KCC2 in the superficial dorsal horn (SDH) of the spinal cord, weakening spinal GABA_A_/glycine-mediated postsynaptic inhibition. Here, we tested whether the administration of small molecules enhancing KCC2, CLP257 and its pro-drug CLP290, may counteract MIH. MIH was typically expressed within 6–8 days of morphine treatment. Morphine-treated rats exhibited decreased withdrawal threshold to mechanical stimulation and increased vocalizing behavior to subcutaneous injections. Chloride extrusion was impaired in SDH neurons measured as a depolarizing shift in *E*_GABA_ under Cl^−^ load. Delivering CLP257 to spinal cord slices obtained from morphine-treated rats was sufficient to restore Cl^−^ extrusion capacity in SDH neurons. *In vivo* co-treatment with morphine and oral CLP290 prevented membrane KCC2 downregulation in SDH neurons. Concurrently, co-treatment with CLP290 significantly mitigated MIH and acute administration of CLP257 in established MIH restored normal nociceptive behavior. Our data indicate that enhancing KCC2 activity is a viable therapeutic approach for counteracting MIH. Chloride extrusion enhancers may represent an effective co-adjuvant therapy to improve morphine analgesia by preventing and reversing MIH.

## Introduction

Paradoxical morphine-induced hyperalgesia (MIH) is a form of nociceptive sensitization in which subjects exposed to morphine treatment develop a paradoxical increased pain sensitivity or exacerbate pre-existing pain^1, 2^. The phenomenon has received increasing attention in clinical settings where it is often referred to in terms of diffuse pain sensation or allodynia in areas unrelated to the pain site for which the morphine treatment was prescribed^2–4^. The overall increase in pain sensitivity diminishes morphine analgesia thus limiting the long-term use of morphine in chronic pain patients. Importantly, MIH has clinical features clearly distinct from tolerance and withdrawal^3^. For example, while increasing morphine doses alleviates tolerance, the same approach was found ineffective or even counterproductive in targeting MIH^5, 6^.

Recent advances have helped unravel the molecular and neuronal mechanisms underlying MIH. Although some investigators have referred to MIH in association with morphine tolerance or withdrawal, it is now well accepted that they are sustained by distinct biological processes. For example, in contrast to MIH, tolerance was associated with platelet-derived growth factor receptor-β receptor signaling^7^ and a recent report identifies microglial pannexin-1 overexpression as a distinct substrate for withdrawal to morphine^8^.

We recently identified a microglia-to-neuron pathway in the spinal dorsal horn which specifically causes MIH, without affecting morphine tolerance^9^. Interestingly, the uncovered molecular pathway recapitulates the mechanistic sequelae observed in nerve-injury models^10^, suggesting a commonality of mechanisms between MIH and neuropathic pain^9, 11^. The molecular cascade involved a P2X4 receptor-dependent release of brain-derived neurotrophic factor (BDNF) from activated microglia which, in turn, down-regulated the K^+^-Cl^−^ co-transporter KCC2 in nociceptive neurons of the superficial dorsal horn (SDH)^9^. Impaired KCC2 activity causes Cl^−^ accumulation, weakening the strength of GABA_A_/glycine receptor-mediated inhibition^12–14^. Disinhibition in the SDH due to decreased KCC2 function translates behaviorally into an increased pain sensitivity, that has been consistently reported in a number of different pain models^9, 13, 15–20^.

Since the down-regulation of KCC2 gates pain hypersensitivity following morphine-treatment, targeting KCC2 appears as a viable strategy to counteract MIH^21^. Recently, we conducted a high-throughput screen to identify and develop novel compounds enhancing KCC2 activity that eventually lead to the development of CLP257 in the arylmethylidine family^22^. In the present study, we tested the efficacy of CLP257, and its carbamate prodrug CLP290, in counteracting MIH. Our data demonstrate that restoring KCC2 expression on the membrane of SDH neurons and accordingly reversing impaired Cl^−^ extrusion capacity in morphine-treated rats can effectively counteract MIH.

## Results

### CLP257 restores Cl^−^ extrusion capacity in SDH neurons from morphine-treated rats

**Figure 1:**
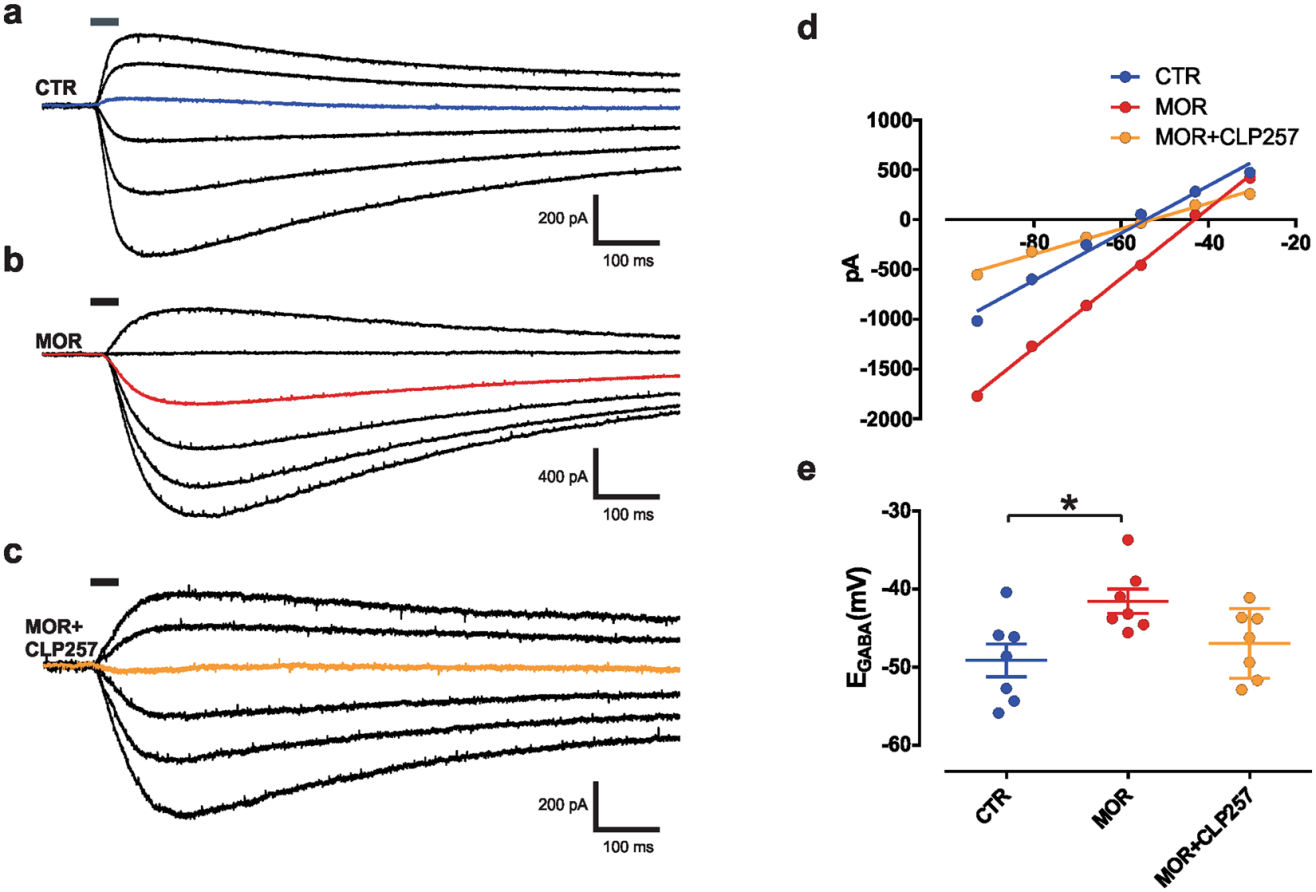
Acute CLP257 restores Cl^−^ extrusion in SDH neurons of morphine-treated rats. (**a**–**c**) Voltage clamp responses at different holding potentials to 30-ms muscimol puffs (*solid line*) in SDH neurons following saline (**a**) or morphine (**b**) treatments in the presence of a Cl^−^ load (29 mM) to measure Cl^−^ extrusion capacity. In (**c**), muscimol response in a SDH neuron from a morphine-treated rat obtained after slice pre-incubation with CLP257 (100 µM) for 1 hour. The colored line indicates the response at −55.5 mV (note the change in polarity). (**d**) *I*-*V* relationships for GABA_A_ currents obtained from neurons in (**a**–**c**). (**e**) Pooled *E*_GABA_ in controls (*n* = 7 cells, *blue*), in morphine-treated rats (*n* = 7 cells, *red*) and following CLP257 treatment (*n* = 7 cells, *orange*). *E*_GABA_ is significantly more depolarized following morphine treatment as compared to control rats (one-way ANOVA, *P* = 0.02, Tukey post-hoc *P = 0.02), but not after CLP257 pre-incubation (*P* = 0.7); MOR *vs*. MOR + CLP (*P* = 0.1). Abbreviations: CTR = control; MOR = morphine; SDH = superficial dorsal horn.

Morphine reduces KCC2 activity in SDH neurons which in turn affects Cl^−^ extrusion capacity^9^. To test whether CLP257 can rescue impaired Cl^−^ extrusion capacity following MIH, we recorded *E*_GABA_ under Cl^−^ load condition (29 mM Cl^−^ in the recording pipette; Fig. 1a–c)^9, 12, 22, 23^. From each recorded neuron, the whole-cell current-voltage relationship (I-V curve) for GABA_A_-activated currents was obtained to allow *E*_GABA_ estimation (Fig. 1d). No differences were observed in the I-V curve slopes (CTR: 20.1 ± 1.6 pA/mV; MOR: 20.7 ± 1.6 pA/mV; MOR + CLP257: 21.8 ± 2.2 pA/mV; n = 6 per group; One-way ANOVA, P = 0.8). However, *E*_GABA_ of SDH neurons in slices from morphine-treated rats was more depolarized as compared to control rats, indicating a reduced Cl^−^ extrusion capacity (Fig. 1e). Pre-incubation of slices, taken from morphine treated rats, for 1 hour with CLP257 (100 µM) restored a normal *E*_GABA_ in SDH neurons (Fig. 1e; one-way ANOVA, P = 0.02).

### Concurrent morphine treatment with the CLP257 prodrug CLP290 prevents KCC2 downregulation in the SDH

**Figure 2:**
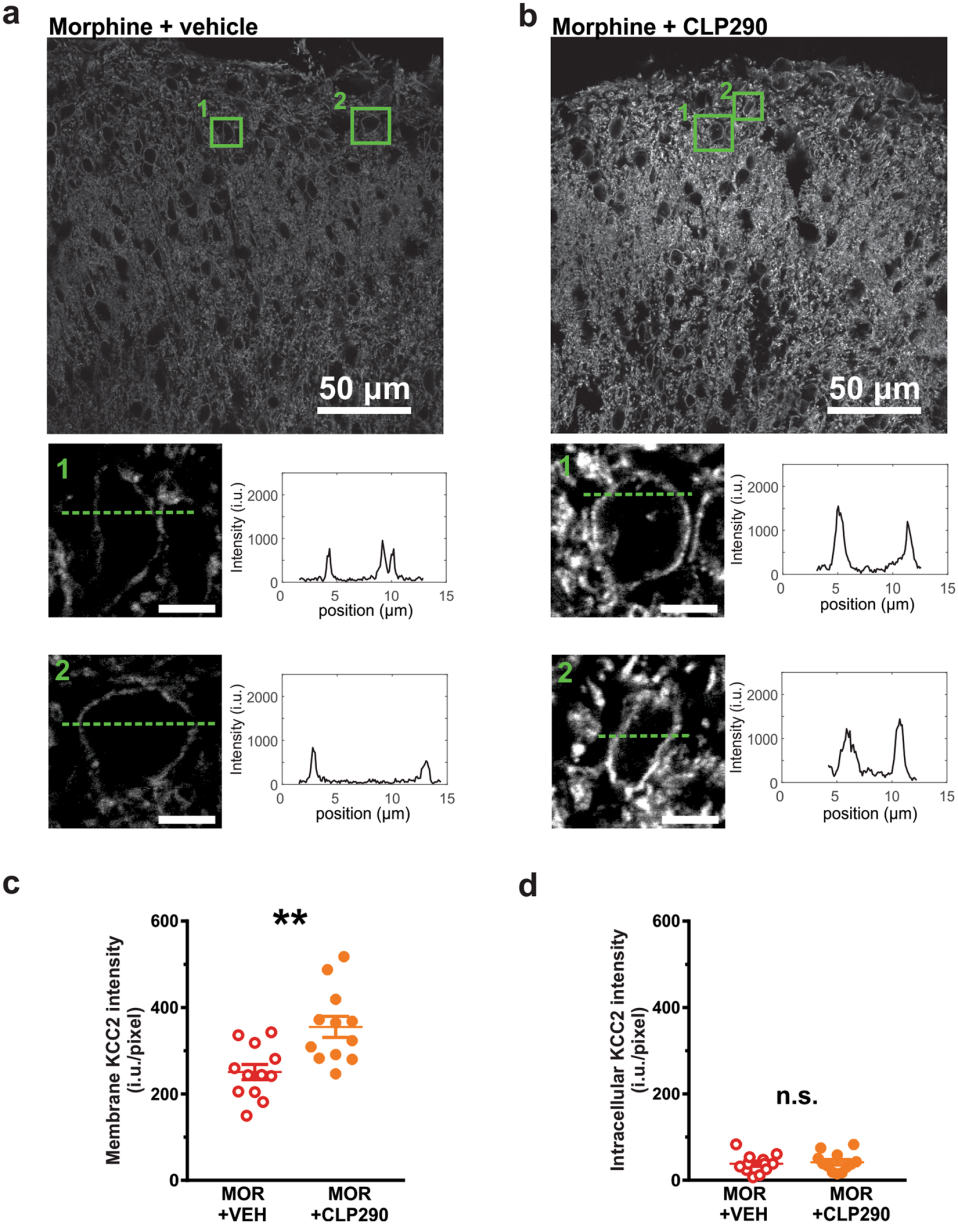
Effect of repeated morphine and CLP290 co-treatment on KCC2 expression. (**a**,**b**) KCC2 expression in the SDH of MOR + VEH and MOR + CLP290 treated rats. The enlargements below show the main membrane localization of KCC2 staining in representative cell bodies of the SDH. (**c**,**d**) Expression of membrane KCC2 (intensity per pixel) was significantly higher in MOR + CLP290 rats (n = 12) as compared to MOR + VEH rats (n = 12, t-test, **P = 0.003). The difference in intensity per pixel was significant for the membrane compartment (**c**; t-test, **P = 0.002) but not the intracellular compartment (**d**, t-test, *P* = 0.4). Abbreviations: VEH = vehicle (20% HPβCD); MOR = morphine; SDH = superficial dorsal horn; n.s. = not significant.

We previously showed that chronic morphine decreases KCC2 expression in the SDH of the rat spinal cord^9^. We therefore wanted to address whether concurrent systemic administration of the CLP257 carbamate prodrug CLP290 (100 mg/kg^22^) could prevent morphine-induced downregulation of spinal KCC2. We found that oral administration of CLP290 given concurrently with morphine (10 mg/kg subcutaneous - s.c. - twice a day) for 7 days prevented the downregulation of KCC2 in the SDH (Fig. 2a and b). A significantly greater level of KCC2 was observed on the membrane compartment in the morphine + CLP290 compared to the morphine-only group (Fig. 2c; MOR + VEH, n = 12; MOR + CLP290, n = 12; t-test, P = 0.002), while no difference was observed in the intracellular compartment (Fig. 2d; P = 0.4), consistent with previous findings^22, 24^.

### Concurrent morphine and CLP290 treatment prevents MIH

**Figure 3:**
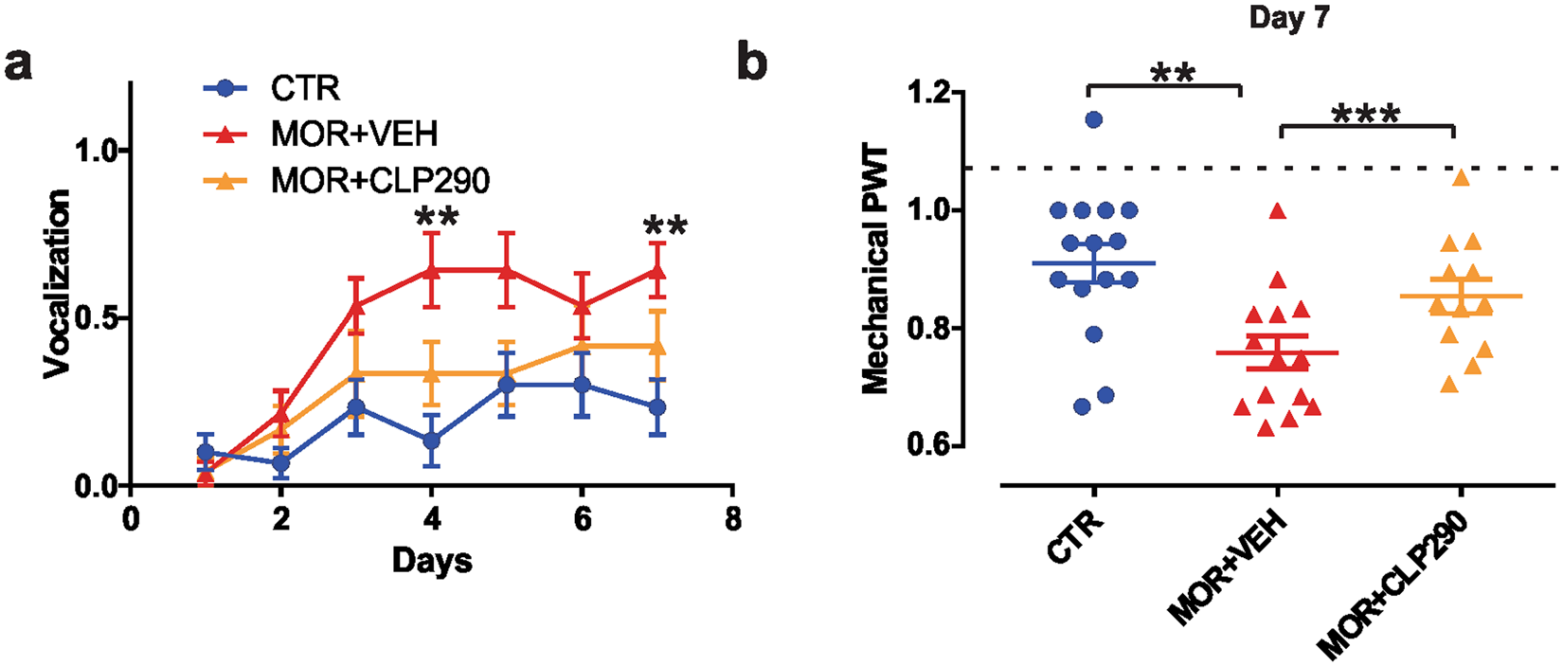
Effect of systemic CLP290 co-treatment on the development of MIH. (**a**) Effect of CLP290 on injection syringe puncture-induced vocalization in CTR (n = 15), MOR + VEH (n = 14) and MOR + CLP290 (n = 12; Kruskall-Wallis test; day 7: *P* = 0.009; CTR *vs*. MOR + VEH, **P < 0.01). (**b**) Effect of CLP290 (100 mg/kg) on paw withdrawal threshold (PWT) in rats treated with morphine for 7 days. CLP290 (n = 12) or VEH (n = 14) was orally administered every day, twice a day, together with s.c. morphine injection for 7 days; data are compared with saline-treated controls (one-way ANOVA, ***P < 0.001). All PWT values are normalized to the baseline. Abbreviations: CTR = control; VEH = vehicle (20% HPβCD); MOR = morphine; PWT = paw withdrawal threshold.

Repeated morphine administration (10 mg/kg s.c. - twice a day) induced an increase in the number of vocalization caused by s.c. injections and an increase in mechanical sensitivity within about one week (Fig. 3a and b, n = 14), as previously reported^9^. CLP290 delivered orally twice a day (100 mg/Kg) concurrently to morphine treatment significantly mitigated MIH in morphine-treated rats (n = 12). That is, CLP290 co-treated rats maintained minimal vocalizing behavior to subcutaneous injections (Fig. 3a; Kruskall-Wallis test, day 7, P = 0.009) and showed a significantly greater nociceptive mechanical withdrawal threshold compared to morphine-only treated rats (day 7, t-test, P < 0.001; Fig. 3b).

### Acute CLP257 treatment reverses established MIH

**Figure 4:**
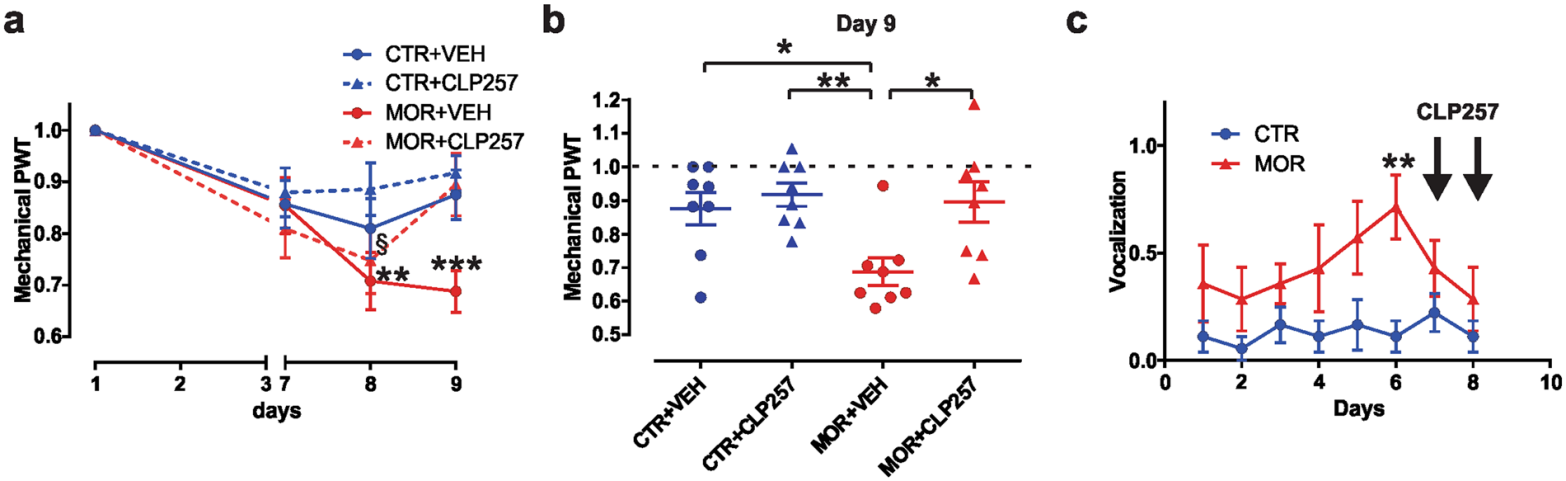
Effect of CLP257 on established MIH. (**a**) Effect of CLP257 on PWT in saline- (blue) and morphine-treated (red) rats (*n* = 8 per group). A single dose of CLP257 (100 mg/kg) or VEH were administered i.p. at day 9. Morphine-treated rats developed mechanical sensitivity at day 8 (repeated measure ANOVA; Bonferroni’s post-hoc day 8 *vs*. day 1: MOR + VEH **P < 0.01; MOR + CLP257, ^§^ *P* < 0.05) which was reversed by CLP257 injection at day 9 (Bonferroni’s post-hoc day 9 *vs*. day 1: MOR + VEH ***P < 0.001; MOR + CLP257, *P* > 0.05). (**b**) Between-group comparison of PWT estimates at day 9 of treatments; one-way ANOVA, *P* = 0.002; Tukey post-hoc: **P < 0.01; *P < 0.05. All threshold values are normalized to baseline. (**c**) Effect of CLP257 on injection-induced vocalization in saline- (n = 9) *vs*. morphine-treated rats (n = 7). Morphine treatment induced a significant increase in vocalizing behavior at day 6 (Mann-Whitney test, *P* = 0.008). Following CLP257 injection (i.p., 100 mg/kg; *black arrows* at day 7 and day 8), no differences were observed (day 7, *P* = 0.299; day 8, *P* = 0.47). Abbreviations: CTR = control; VEH = vehicle (20% HPβCD); MOR = morphine; PWT = paw withdrawal threshold.

To test whether CLP257 not only prevents but also acutely reverses established MIH, we decided to deliver the native molecule, rather than its carbamate prodrug, because of the more rapid onset of the antihyperalgesic response^22^. CLP257 was administered by a single intraperitoneal (i.p. – 100 mg/Kg) injection following 8 days of morphine treatment (10 mg/kg s.c. - twice a day; Fig. 4a). CLP257 treatment (n = 8) induced a significant increase in mechanical sensitivity as compared to morphine-treated rats receiving vehicle alone (n = 8) restoring mechanical thresholds comparable to that observed in control saline-treated rats (n = 8; one-way ANOVA, P = 0.002; Fig. 4b). Conversely, CLP257 injection in saline-treated rats did not produce any significant change in mechanical sensitivity (n = 8; P > 0.05). In parallel, we observed that CLP257 treatment also reversed the morphine-induced increase in vocalizing responses to subcutaneous injections (Fig. 4c).

## Discussion

We showed here that CLP257 and its carbamate prodrug CLP290 can respectively rescue established MIH and prevent its development by restoring Cl^−^ transport or preventing its deficiency in the SDH.

The downregulation of KCC2 in the CNS, and ensuing loss of inhibitory strength, has been observed in a variety of neurological diseases which are etiologically associated with an increased neuronal excitability^9, 15, 25–27^. Thus, targeting KCC2 is a promising strategy to restore synaptic inhibition and stabilize the excitatory *vs*. inhibitory imbalance in these pathological conditions^21^. Importantly, KCC2 is only expressed in central neurons^28^, thus systemic administration of KCC2 enhancers has no impact on peripheral neurons nor other non-neuronal tissue. Moreover, in mature neurons, KCC2 already operates near its equilibrium, therefore a pharmacological increase of its activity is unlikely to produce significant adverse effects on excitability under normal conditions^12, 23, 29^. Conversely, since even a small drop in KCC2 activity may have a dramatic impact on the efficacy of inhibition, restoring KCC2 function in neurons with impaired Cl^−^ extrusion capacity can effectively maintain inhibitory control^9, 12, 30^.

The identification of small molecules enhancing KCC2 function has provided proof of principle of the value of rescuing KCC2 in pathological conditions where KCC2 activity is hampered^22^. Here, MIH is added to the list of potential beneficial conditions to treat by rescuing KCC2. Administration of CLP257 to nerve-injured rats was found sufficient to reverse pain hypersensitivity by restoring inhibition in SDH neurons^22^. The efficacy of CLP257 in targeting KCC2 was subsequently confirmed by independent studies in other brain areas^31, 32^, as well as the ability of CLP290 to properly restore inhibition^24, 33^ and rescue impaired KCC2^24^. The precise mechanisms by which these molecules modulate KCC2 activity remain however largely unknown. In our previous study^22^, we showed that a single injection of CLP257 rapidly restored altered nociceptive behavior, while its carbamate prodrug CLP290 with an improved pharmacokinetic profile induced a slow onset but long lasting effect. Here, in conditions where the KCC2 levels are diminished because of morphine chronic treatments^9^, we similarly showed that acute CLP257 administration could rapidly rescue Cl^−^ homeostasis and that sustained administration of CLP290, could prevent membrane KCC2 downregulation in the SDH.

Our data suggest that, by enhancing KCC2 activity and restoring Cl^−^ transport in SDH neurons of morphine-treated rats, CLP257 and CLP290 reversed or prevented MIH, respectively. This is consistent with our previous observations that morphine treatment causes a downregulation of KCC2 and a reduction of Cl^−^ extrusion capacity in SDH neurons, thus dramatically weakening the strength of synaptic inhibition in central nociceptive pathways^9^. Morphine treatment appears to trigger a sequence of molecular and cellular changes mirroring the mechanisms described in neuropathic pain^10^, which may explain the poor efficacy of morphine in relieving neuropathic pain symptoms^11^. In this molecular scenario, KCC2 represents the end point of a complex microglia-to-neuron cascade mediated by P2X4-BDNF-TrkB signaling that specifically gates MIH^9^. Blocking any point of this sequence of signaling events limited MIH without affecting morphine tolerance^9^. Targeting KCC2 emerges as a novel and specific strategy to counteract MIH and to improve the analgesic efficacy of morphine, especially when a chronic exposure to opiates is required. Importantly, not only could MIH be prevented, but the paradoxical hyperalgesia could be reversed, once established. These findings demonstrate that KCC2 enhancers may represent an effective co-adjuvant therapy to improve morphine analgesia by preventing or reversing one of morphine’s most debilitating side effects.

## Methods

### Animals

Adult male rats (300 g, >postnatal day 60) were housed under a 12 h:12 h light/dark cycle. All experimental procedures have been performed in accordance with guidelines from the Canadian Council on Animal Care (CCAC) and approved by the committee for animal protection of Université Laval (CPAUL; authorization number: 2014–031–3 and 2014–027–3).

### Chronic morphine protocol and pharmacological treatments

Morphine sulfate (50 mg/ml) was diluted in saline sterile solution immediately before injection. Morphine or saline were subcutaneously (s.c.) injected twice a day (10 mg/kg; 9 a.m. – 6 p.m.) in naïve adult rats, as previously described^9^. The KCC2 enhancer CLP257 and its carbamate pro-drug CLP290 were freshly diluted in 20% 2-hydroxypropyl-β-cyclodextrin (HPCD) prior injection, as described. CLP257 or vehicle were delivered intraperitoneally (i.p.) after 7–8 days of morphine or saline, as described (100 mg/kg^22^). CLP290 or vehicle were delivered orally by gavage twice a day for the whole duration of the morphine/saline treatment (100 mg/kg^22^).

### Behavioral tests

To be able to detect nociceptive withdrawal threshold unclouded by the analgesic effect of morphine, mechanical sensitivity was tested 1 hour prior to morning morphine or saline s.c. injection at day 1, 3, 7, 8, 9 by applying calibrated von Frey filaments (Bioseb, France). The paw withdrawal threshold (PWT) was estimated by applying a modified “up and down” approach (SUDO method^34^). Briefly, PWT estimate were obtained by adding an adjustment value of ±0.5 stimulus intervals to the log force value of the fifth filament used in each test. The adjustment factor was positive if there was no response to the fifth filament, or negative if there was a withdrawal. When CLP257 or vehicle were delivered i.p., PWT was tested 2 hrs after i.p. injection. Statistical analysis was performed on log stimulus values as previously recommended^35^ and data were subsequently normalized to day1 as described^9^.

Puncture-induced vocalizations were also monitored during s.c. injections of morphine itself and the expression of vocalizing behavior considered as an index of hyperalgesia^9^. Behavioral experimenter was blind to the pharmacological treatments.

### Electrophysiology

After 7 days of s.c. treatment with saline or morphine, rats were sacrificed with an i.p. injection of xylazine/ketamine (8.75/1.25 mg per 100 g) and parasagittal slices (300 µm) of spinal cord were prepared as previously described^36^. Briefly, rats were transcardially perfused with ice cold oxygenated (95%O_2_, 5%CO_2_) artificial cerebrospinal fluid (ACSF) containing the following (in mM): 252 sucrose, 2.5 KCl, 2 MgCl_2_, 2 CaCl_2_, 1.25 NaH_2_PO4, 26 NaHCO_3_, 10 glucose, and 5 kynurenate. After decapitation, spinal cords were removed by hydraulic extrusion and slices were cut in the same ice-cold solution with a vibrating microtome (Leica VT1200 S, Germany). Slices were allowed to recover for 30 min at 34 °C, then moved at room temperature in ASCF containing (in mM): 126 NaCl, 2.5 KCl, 2 MgCl_2_, 2 CaCl_2_, 1.25 NaH_2_PO_4_, 26 NaHCO_3_, 10 glucose. When required, CLP257 (100 μM) was added to ASCF for 1 hour prior to recording.

Voltage clamp recordings were performed from visually identified SDH neurons as previously described^9^. Recordings were performed from SDH neurons located within 50 µm from the dorsal white matter. *E*_GABA_ was measured under Cl^−^ load (29 mM) with a pipette solution containing 115 mM potassium methylsulfate, 25 mM KCl, 2 mM MgCl_2_, 10 mM HEPES, 4 mM Na-ATP, 0.4 mM Na-GTP, pH 7.2. Membrane potential measurements were corrected off-line for liquid junction potential. Data were filtered at 5 kHz, digitized and acquired using pClamp 10.2 software (Molecular Devices, USA). The GABA_A_ agonist muscimol (1 mM) was dissolved into a HEPES-buffered ACSF and applied by brief puffs (30 ms) from a patch pipette. Muscimol responses were obtained in voltage clamp at increasing holding potentials (in 12.5 mV steps) in the presence of TTX (1 µM), APV (40 µM) and CNQX (10 µM). Experimental *E*_GABA_ was interpolated from the GABA_A_ I-V relationships, obtained by averaging 3 responses for each voltage step. The difference between the experimental and the theoretical *E*_GABA_ (According to Hodgkin-Katz-Goldman equation) provided an estimate of Cl^−^ extrusion capacity^9, 37^.

### Immunohistochemistry

*R*ats were anaesthetized with ketamine/xylazine i.p. (8.75/1.25 mg per 100 g) and perfused transcardially with 4% paraformaldehyde in 0.1 M PB (pH 7.4). Spinal cord segments L4-L5 were collected and post-fixed for 60 min in the same fixative and cryoprotected in 30% sucrose in 0.1 M PB overnight at 4 °C. Transverse sections were cut at 25 µm on a sledge freezing microtome Leica SM2000R (Leica Microsystems). Sections were pre-incubated in PBS (pH 7.4) with 0.2% Triton (PBS + T) and 1% normal goat serum (NGS) for 30 min. After washing, sections were incubated overnight at 4 °C with mixture primary antibodies (see below). Then, sections were washed 3 times in PBS + T and then incubated for 2 hours with secondary antibody (1:500; highly purified Cy3 Goat Anti-Rabbit IgG, Jackson ImmunoResearch laboratories Inc. Catalog#111–165–144). Sections were dehydrated and mounted on antifade mounting medium (Dako, Japan).

A rabbit anti-KCC2 antibody was used (1:1000; Millipore-Upstate, USA, catalog #07–432). This immunogen is highly specific for the rat KCC2 and does not share any homology with other cation-Cl^−^ cotransporters^38^.

### Confocal laser scanning microscope

Images (12-bits, 2048 × 2048 pixels, pixelsize = 0.103 µm) were obtained with an Olympus FV1000 (Olympus America Inc., USA) confocal laser scanning microscope (CLSM) with a 60X plan-apochromatic oil immersion objective (NA 1.4) using dichroic filter FV-FCBGR 488/543/633. For KCC2 fluorescently tagged with Cy3 imaging, a 543 nm laser (HeNe) was used for excitation together with a 605 nm bandpass emission filter for collection. Optimal laser settings were chosen to minimize saturation and photobleaching. For quantification, all CLSM settings were then kept constant for acquisition across all samples. For sake of comparison, the contrast and intensity range (0 to 2000 i.u) for the images presented (Fig. 2a and b) were kept constant.

### Membrane KCC2 index analysis

SDH corresponds to a complex dense network of cells in which the majority of single cells cannot be clearly separated or delineated. As can be observed from single cell bodies in the confocal images (Fig. 2a and b), the highest density of KCC2 fluorescence signal is at the cell membrane. Nevertheless, the cell membrane volume only corresponds to a small fraction of the total volume. Indeed, the cell membrane is roughly ~10 nm thick which is small compared to the diameters of dendrites (~0.2–2.0 µm) and cell bodies (>5 µm). Moreover, even if one could perfectly delineate the cell membrane, the measurement would still be heavily tainted by the presence of intracellular KCC2 due to the optical resolution as determined by the point spread function (PSF). Theoretical PSF of the confocal microscope (NA = 1.4, 1 Airy unit pinhole size, λ_Excitation_ = 543 nm and λ_Emission_ = 605 nm) can be approximated as a three dimensional Gaussian with full width half maximum (FWHM) diameters of 200 nm for lateral and 400 nm for axial resolution. The membrane thus only represents a small portion of the PSF. Many super-resolution microscopy approaches were developed to achieve sub-100 nm resolutions^39, 40^. Nevertheless, even if resolution enhancement in dense biological samples can be achieved on small spatial fields for specific cases^41, 42^, the issue of not sampling exclusively from the cell membrane, and hence including intracellular signal, is still present and has to be considered when trying to estimate the membrane signal.

Because dendritic KCC2^23^ plays a critical role in overall Cl^−^ transport, we sought to define an index that reflects total membrane KCC2 intensity, not just easily delineatable cell body membrane (Fig. 2a and b). For each image of an immunostained slice, the average non-specific staining level (per pixel), quantified in isolated white matter regions, was first subtracted from the whole image. The intensity of the intracellular KCC2 immunostaining was then defined in regions that could undoubtedly be identified as neurons of the SDH. Finally, a large region of the superficial dorsal horn (<70 µm from the edge of the white mater) was delineated from which the averaged pixel intensities was calculated. To obtain the KCC2 intensity corresponding to the membrane staining, the average per pixel intracellular KCC2 intensity value was subtracted from the average per pixel intensity of the whole KCC2 in the chosen region. This resulting membrane KCC2 index was considered robust and global because it includes many neuronal cell bodies and dendrites and does not depend on arbitrarily visually selected neurons.

The membrane KCC2 index was measured for every rat and the values were averaged using four to six spinal cord sections per rat in both conditions (n = 12 rats for morphine + vehicle; n = 12 rats for morphine + CLP290). Single profile plots of the KCC2 immunostaining intensities in identified dorsal horn neurons are also shown in Fig. 2a and b for comparison.

### Statistical analysis

Statistical analysis was performed with GraphPad Prism 7 (GraphPad Software; USA). Differences were analyzed by t-test or by one-way analysis of variance (ANOVA) followed by Tukey post-hoc test for multiple comparisons. Values obtained from the same subjects at different time points were analyzed with repeated-measure ANOVA and Bonferroni post-hoc. Dataset with non-normal distribution (percentages of vocalizing rats) were analyzed by appropriate non-parametric tests (Mann-Whitney and Kruskall-Wallis as indicated).

Electrophysiological data were reported as mean ± SEM, with *n* indicating the number of recorded neurons. Histological and behavioral measurements were expressed as mean ± SEM, with *n* indicating the number of rats. Values of *P* < 0.05 were considered statistically significant.
